# Osteopontin Serum Concentration and Metabolic Syndrome in Male Psoriatic Patients

**DOI:** 10.3390/jcm10040755

**Published:** 2021-02-13

**Authors:** Joanna Bartosińska, Joanna Przepiórka-Kosińska, Beata Sarecka-Hujar, Dorota Raczkiewicz, Małgorzata Kowal, Katarzyna Chyl-Surdacka, Jarosław Bartosiński, Jakub Kosiński, Dorota Krasowska, Grażyna Chodorowska

**Affiliations:** 1 Department of Cosmetology and Aestetic Medicine, Medical University of Lublin, ul. Chodźki 1, 20-093 Lublin, Poland; 2 Department of Dermatology, Venereology and Pediatric Dermatology, Medical University of Lublin, ul. Staszica 11, 20-081 Lublin, Poland; asia880124@wp.pl (J.P.-K.); kowalma71@o2.pl (M.K.); kasiachyl@gmail.com (K.C.-S.); dorota.krasowska@umlub.pl (D.K.); grazyna.chodorowska@umlub.pl (G.C.); 3 Department of Basic Biomedical Science, Faculty of Pharmaceutical Sciences in Sosnowiec, Medical University of Silesia in Katowice, ul. Kasztanowa 3, 41-200 Sosnowiec, Poland; bsarecka-hujar@sum.edu.pl; 4 Department of Medical Statistics, School of Public Health, Center of Postgraduate Medical Education, ul. Kleczewska 61/63, 01-826 Warsaw, Poland; dorota.bartosinska@gmail.com; 5 Department of Anaesthesiology and Intensive Therapy, Independent Public Clinical Hospital No. 4 in Lublin, ul. Jaczewskiego 8, 20-954 Lublin, Poland; jeremybartosinski@gmail.com; 6 Department of Rehabilitation and Orthopedics, Medical University of Lublin, ul. Jaczewskiego 8, 20-954 Lublin, Poland; kuba.kosinski@gmail.com

**Keywords:** psoriasis, metabolic syndrome, osteopontin

## Abstract

Psoriasis (Ps) is an immune-mediated inflammatory skin disease that is widely associated with the clinical features of metabolic syndrome (MetS), including hypertension, abdominal obesity, insulin resistance, type 2 diabetes and dyslipidemia. Osteopontin (OPN), a multifunctional protein involved in the modulation of inflammatory processes, may contribute to the development of atherosclerosis and MetS. Therefore, the aim of the study was the assessment of the correlation between OPN concentration in the peripheral blood and the presence of MetS as well as its particular components in the Ps patients. The study comprised 107 male Ps patients (50 patients with MetS and 57 without MetS) and 38 healthy volunteers (HVs). The concentration of OPN in serum was determined using enzyme-linked immunosorbent assay (ELISA) method. Fasting blood glucose and lipid profile components: total cholesterol (total CHOL), high-density lipoprotein cholesterol (HDL-CHOL), low-density lipoprotein cholesterol (LDL-CHOL), triglycerides (TG) were examined. Ps patients with MetS had significantly higher obesity, systolic blood pressure, TG, CHOL/HDL, LDL/HDL and TG/HDL ratios than Ps patients without MetS. OPN serum concentration was significantly higher in the Ps patients than in the HVs (*p* = 0.022) but not significantly different between the Ps patients with and without MetS (*p* = 0.275). OPN serum concentration in Ps patients correlated negatively with total CHOL (*p* = 0.004) and TG (*p* = 0.009). OPN is increased in Ps patients and may serve as a biomarker of some lipid abnormalities in them.

## 1. Introduction

Psoriasis (Ps) is a chronic inflammatory skin disease of complex pathogenesis with genetic predisposition, environmental factors and immunological disturbances. Chronic inflammation, which is the essence of pathophysiological phenomena in Ps, is also a factor predisposing these patients to the occurrence of systemic disorders, including diabetes, hypertension, lipid disorders and cardiovascular diseases (CVDs). Numerous inflammatory mediators produced by various cells, including Th—Th1, Th17, Th22 subpopulations, which produce such cytokines as tumor necrosis factor α (TNF-α), interleukin 17 (IL-17), IL-22, participate in the formation of psoriatic plaques as well as in the development of atherosclerosis and CVDs [1,2].

The increased risk of hypertension, abdominal obesity, insulin resistance and dyslipidemia in Ps patients contributes to the development of metabolic syndrome (MetS) [1,3,4]. The probability of obesity in Ps patients is almost twice as high as in the general population [3,4]. Moreover, it has been demonstrated that with the increase in body mass index (BMI), the risk of Ps increases [4], and the increased production of TNF-α in obese patients shows a positive correlation with the amount of IL-17 produced [5]. Both obesity and systemic inflammation contribute to the development of insulin resistance and type 2 diabetes [4]. Patients with Ps have an increased risk of dyslipidemia, i.e., elevated triglycerides, LDL (low-density lipoprotein) cholesterol, VLDL (very-low-density lipoprotein) and decreased HDL (high-density lipoprotein) cholesterol [2,3,4].

The results of many studies indicate that an increased risk of myocardial infarction may be observed in Ps patients who do not have traditional risk factors [1,2,3,4]. It is suggested that in Ps patients a faster development of atherosclerosis is observed [5]. The occurrence of other CVDs that may be associated with Ps, including atrial fibrillation, additionally increases the risk of ischemic stroke, coronary heart disease and sudden cardiovascular death [4]. Similar to myocardial infarction, the risk of stroke is particularly high in patients with severe Ps and is independent of other known risk factors for stroke [6,7].

The previous data indicated that osteopontin (OPN) participated in both acute and chronic inflammatory processes [8]. At the inflammatory site, OPN is secreted mainly by T lymphocytes and activated macrophages, whereas circulating monocytes do not have this property. OPN also stimulates the chemotaxis of macrophages and the secretion of IL-12 as well as promotes the chemotaxis and the adhesion of T lymphocytes and their proliferation [8,9,10,11,12,13,14]. It has been found that low concentration values or the lack of expression of OPN leads to the reduction of inflammation [15]. OPN also contributes to the formation of atherosclerotic plaque [11,14,16]. In damaged blood vessels, OPN takes part in their repair and remodeling by increasing proliferation and migration of smooth muscle cells and endothelium [10,11,14]. OPN is also an important regulator of calcification and vascular mineralization [11,14]. It was found that the increased concentration of OPN was associated with a higher risk of developing CVDs, regardless of the presence of traditional risk factors [11]. Increased plasma OPN levels in patients with coronary artery disease and chronic heart failure suggest that it may be a new marker of CVDs [14]. OPN is believed to be involved in the development of obesity, insulin resistance and type 2 diabetes [10,17,18]. In the course of diabetes, OPN takes part in the development of changes in blood vessels by influencing the nuclear factor of activated T cells. Both obesity and type 2 diabetes coexist with non-alcoholic fatty liver disease, which may be related to the observed increase in OPN levels in hepatocytes [1,2,17].

The participation of OPN, a pro-inflammatory, angiogenic and anti-apoptotic protein, may be significant in Ps pathogenesis, contributing to the occurrence of chronic inflammation and thus to the development of coexisting diseases, such as obesity, diabetes, insulin resistance, metabolic disorders, atherosclerosis and other CVDs.

Therefore, the aim of the study was the assessment of the correlation between OPN concentration in the peripheral blood and the presence of MetS as well as its particular components.

## 2. Materials and Methods 

### 2.1. The Study Groups

The study comprised 107 male Ps patients and 38 age-matched healthy male volunteers (HVs). The Ps patients and HVs underwent physical examination, including the measurement of body weight, height, waist and hip circumference, arterial pressure and heart rate. Body mass index (BMI) and waist-to-hip ratio (WHR) were calculated in Ps patients and HVs.

The exclusion criteria were female sex, treatment with topical and/or systemic anti-psoriatic medications less than 3 months before the recruitment into the study, history of malignancy and anti-cancer treatment.

The inclusion criteria were male sex, psoriasis diagnosis confirmed by dermatologist.

Female psoriatic patients were excluded due to the possible influence of sexual hormones, i.e., estrogens on *OPN* gene [19]. Moreover, there are some observations that OPN levels differ in pre- and postmenopausal women and may be associated with bone loss and osteoporosis [20,21].

Informed consent was obtained from each individual. The study protocol complies with the ethical guidelines of the 1975 Declaration of Helsinki. The study was approved by the local ethics committee (KE-0254/30/2016).

### 2.2. Characteristics of Psoriasis in the Studied Ps Patients

Medical history, including the duration of Ps, family history and co-morbidities was taken. The severity of Ps was assessed using Psoriasis Area and Severity Index (PASI), Body Surface Area (BSA) and Physician Global Assessment (PGA).

The Ps patients who had not received any topical and systemic anti-psoriatic treatment for at least 3 months before the recruitment were included in the study.

The age of Ps onset ranged from less than 1 year to 65 years, with an average of 25 years. Psoriasis duration was from a few months to 48 years, 19 years on average. In the studied patients, the severity of Ps skin lesions expressed by PASI was between 3 and 45 with an average of 13. The extent of Ps skin lesions expressed by BSA in the studied patients was from 5% to 90%, 25% on average. PGA was minimal in 13 patients (12.15%), mild in 57 patients (55.74%), moderate in another 35 individuals (32.71%) and severe in 2 patients (1.87%).

### 2.3. Assessment of Serum OPN Concentration in Ps Patients and HVs

In Ps patients and HVs, peripheral blood samples were collected. The blood samples were centrifuged for 15 min at 1000× *g* and stored at −80 °C until tested. The concentration of OPN in serum was determined using enzyme-linked immunosorbent assay (ELISA) kit (Quantikine^®^ELISA Human Osteopontin, R&D Systems, Inc., Minneapolis, MN, USA).

### 2.4. Determining the Presence of MetS in Ps Patients and HVs

In the analyzed groups, the following laboratory tests were performed: fasting blood glucose and lipid profile components: total cholesterol (total CHOL), HDL cholesterol (HDL-CHOL), LDL cholesterol (LDL-CHOL), triglycerides (TG). The presence of MetS was defined as the coexistence of at least 3 out of the 5 following risk factors:

1. Waist circumference ≥ 94 cm,
2. TG ≥ 150 mg/dL or treatment for dyslipidemia,
3. HDL-CHOL < 40 mg/dL or treatment for dyslipidemia,
4. Systolic blood pressure ≥ 130 mmHg and/or diastolic blood pressure ≥ 85 mmHg or antihypertensive therapy,
5. Fasting glucose ≥ 100 mg/dL or hypoglycemic treatment.

MetS was found in 50 Ps patients, i.e., 46.73%, while it was not present in HVs. In the group of Ps patients with MetS, 11 cases (22%) were treated for dyslipidemia with the use of statins.

### 2.5. Statistical Methods

The data were statistically analyzed using STATISTICA 13 software. The minimum and maximum values and the median and interquartile range (25–75%) were estimated for continuous variables, as well as absolute numbers (*n*) and percentages (%) of the occurrence of items for categorical variables.

The following statistical tests were used:

- Pearson’s chi-square test to compare categorical variables between psoriatic patients and healthy volunteers, between psoriatic patients with and without metabolic syndrome;
- Mann–Whitney’s U test to compare: continuous variables between two groups, OPN between two categories of categorical variables;
- Kruskal–Wallis’s H test to compare continuous variables between three groups, OPN between more than two categories of categorical variables;
- Spearman’s correlation coefficient to correlate OPN with continuous variables.
- The significance level was assumed as 0.05.

## 3. Results

### 3.1. Characteristics of Ps Patients and HVs

The studied Ps patients’ ages ranged from 18 to 77 years, 47 years on average. The average age of the HV group was 48 years and did not significantly differ from the age of Ps patients (*p* = 0.953).

Overweight and obesity were significantly more common in Ps patients (35.51% and 34.58%, respectively) in comparison to HVs (15.79% and 0.00%, respectively), *p* < 0.001. WHR of at least 1 was found in 41.12% of Ps patients, whereas in all HVs WHR was within normal limits. Systolic and diastolic blood pressure, total CHOL and TG serum concentrations, CHOL/HDL, and TG/HDL ratios were significantly higher in Ps patients than in HVs (*p* < 0.05). However, LDL-CHOL and glucose levels did not differ significantly between Ps patients and HVs (*p* = 0.291 and *p* = 0.817, respectively) (Table 1).

### 3.2. Comparison of Ps Patients with and without MetS

The characteristics presented in Table 1 differed significantly between Ps patients with MetS vs. Ps without MetS vs. HVs, except for LDL-CHOL and glucose serum concentration. Ps patients with MetS had significantly higher BMI, WHR, waist circumference, systolic blood pressure, TG, CHOL/HDL, LDL/HDL and TG/HDL ratios than Ps patients without MetS. However, diastolic blood pressure, total CHOL and LDL-CHOL did not differ significantly between Ps patients with and without MetS.

### 3.3. Comparison of OPN between Ps Patients and HVs as Well as between Ps Patients with and without MetS

**Figure 1:**
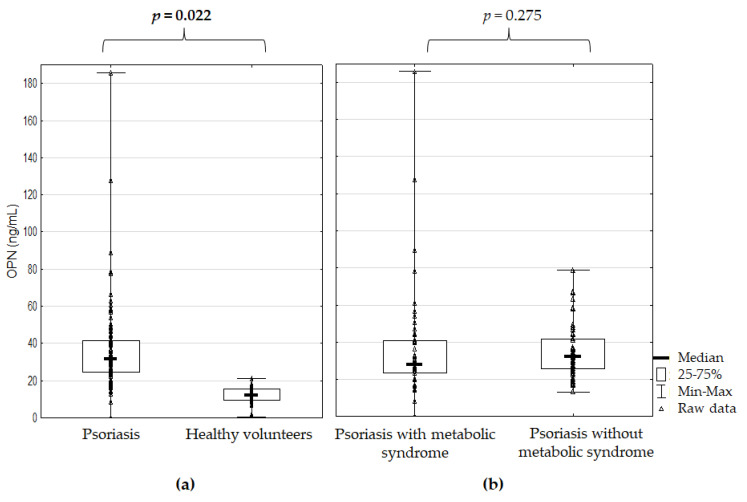
Osteopontin serum concentration (**a**) in the psoriatic patients versus healthy volunteers and (**b**) in the psoriatic patients with and without metabolic syndrome; *p* for Mann–Whitney’s U test; significant difference is in bold.

OPN serum concentration was significantly higher in the Ps patients than in the HVs (*p* = 0.022) but not significantly different between the Ps patients with and without MetS (*p* = 0.275) (Figure 1).

### 3.4. Correlations between OPN and Ps Patients’ Characteristics

OPN serum concentration did not correlate with BMI, WHR, waist circumference, systolic and diastolic blood pressure, glucose, LDL–CHOL and HDL–CHOL concentrations as well as CHOL/HDL and LDL/HDL ratios in Ps patients (Table 2).

**Figure 2:**
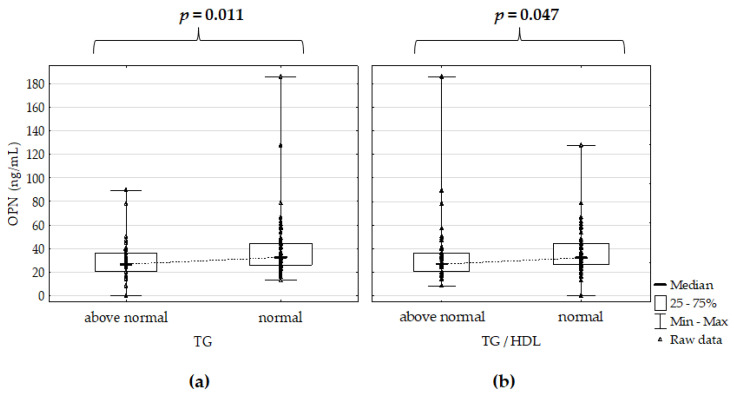
Osteopontin serum concentration (**a**) versus intervals of triglycerides (TG) and (**b**) triglycerides/high–density lipoprotein (TG/HDL) ratio in the psoriatic patients; *p* for Mann–Whitney’s U test; significant differences are in bold.

However, OPN serum concentration in Ps patients correlated negatively with total CHOL (*r* = –0.274, *p* = 0.004) and TG (*r* = –0.250, *p* = 0.009). OPN serum concentration was significantly lower in Ps patients, with TG and TG/HDL ratio above standards in comparison to normal ranges (Figure 2).

### 3.5. OPN versus MetS Components in Ps Patients

OPN serum concentration was significantly lower in Ps patients with hypertriglyceridemia in comparison to Ps patients without hypertriglyceridemia (Table 3). However, OPN serum concentration did not correlate to prevalence of abdominal obesity, low HDL–CHOL, arterial hypertension and hyperglycemia in Ps patients (Table 3).

## 4. Discussion

In the present study we aimed to assess the concentration of OPN regarding the presence of individual components of MetS. We observed no significant correlations between OPN levels and obesity indices such as BMI (*p* = 0.916), WHR (*p* = 0.227) and waist circumference (*p* = 0.280) in the studied Ps patients. 

Previously, a high level of OPN together with an increased amount of C–reactive protein (CRP) in atherosclerotic plaques was observed [22]. Both proteins may act synergistically in various inflammatory processes, including atherosclerosis; however, the exact mechanisms of interaction between CRP and OPN are still unknown. Yan et al. [23] demonstrated that plasma OPN concentration positively correlates with the severity of coronary artery disease in type 2 diabetes patients, which is independent of the occurrence of conventional cardiovascular risk factors.

In the course of Ps, increased triglycerides, LDL cholesterol and decreased HDL cholesterol values are the most frequently observed lipid disturbances [1,2]. In our study, the levels of total CHOL and TG as well as CHOL/HDL and TG/HDL ratios were significantly higher in Ps patients than in HVs, while LDL–CHOL did not differ between both groups. In the Ps patients, we observed a negative correlation between OPN levels and total CHOL as well as TG. OPN serum concentration did not correlate with LDL–CHOL and HDL–CHOL concentrations as well as CHOL/HDL and LDL/HDL ratios in the patients. 

The OPN level was found to be significantly lower in Ps patients with MetS and hypertriglyceridemia than in Ps patients without MetS. Since 11 Ps patients with MetS (22%) were treated with statins, this might explain the study results.

Similar to the results of our study, Kadry et al. [24] did not observe any correlation between plasma concentration of OPN and waist circumference, whereas Toossi et al. [25] did not find a correlation between the plasma concentration of OPN and BMI. Despite higher OPN levels in Ps patients, Duarte et al. [26] did not show any significant differences in OPN levels in obese Ps patients compared to those with normal body weight. Similar to our analyzed group, other studies concerning obese patients demonstrated elevated plasma OPN values [27,28,29,30]. However, no significant differences in OPN concentrations were reported when overweight and obese patients were compared [30].

Contrary to the present study, Robati et al. [31] observed a positive correlation between plasma OPN concentration and BMI in Ps patients. 

Some studies indicated that OPN is involved in pathophysiological processes leading to the development of type 2 diabetes, especially when the disease co–occurred with atherosclerosis [13]. Yan et al. [23] found that the level of OPN is proportional to the severity of nephropathy and coronary heart disease in patients with type 2 diabetes. The authors suggested that OPN could be used as an indicator to evaluate the degree of diabetic vasculopathy [23]. Cai et al. [32] demonstrated that glucose can induce histone acetylation and methylation, which leads to upregulation of expression of *OPN* gene. Thus, by inhibiting histone methyltransferase, the deleterious effect of glucose can be reversed. 

The study by Chen et al. [33] observed higher plasma OPN concentration in Ps patients with hypertension and diabetes. According to some authors, hypertension and chronic renal failure seem to be related to the level of OPN [13]. In patients with hypertension, Kurata et al. [22] found not only a positive correlation of OPN with CRP but also with aldosterone, which stimulates kidney fibroblasts secretion of OPN and collagen. Moreover, according to some reports, the concentration of OPN is associated not only with risk factors of CVDs, such as increased systolic and diastolic blood pressure, but also with higher BMI values and decreased HDL cholesterol [34,35].

Kadry et al. [24] also did not report a correlation between the value of OPN concentration in plasma and the presence of dyslipidemia. In the patients with hypercholesterolemia treated with atorvastatin, Tanaka et al. [36] observed significantly reduced plasma levels of OPN. The authors suggested that, among others, the beneficial effect of statins on CVDs may result from decreasing the OPN concentration in peripheral blood. In opposition to our study results, Robati et al. [31] demonstrated significant positive correlations between OPN concentration and total cholesterol as well as triglyceride levels in their Ps patients. The authors also found a positive correlation between levels of OPN in plasma and intima media wall thickness of the common carotid artery in the Ps patients compared to the control group.

It is suggested that the association between Ps and MetS may result from adipocyte dysfunction, chronic elevated levels of free fatty acids and increased levels of inflammatory cytokines (such as TNF–α and IL–6) which have a systemic effect on insulin regulation and lipid metabolism [37]. Such an association between Ps and MetS is a possible explanation of the mechanism of inflammatory march leading to the development of Ps co–morbidities [37]. Kadry et al. [24] found significantly higher values of plasma OPN concentration in Ps patients with MetS than in the control group. Another study by Kadry et al. [38] showed a positive correlation between the incidence of MetS and the PASI value. The authors believe that this may indicate common pathophysiological pathways of inflammation in both Ps and MetS. Abdel Hay et al. [39] showed significantly higher concentration of OPN in Ps patients compared to the control group as well as higher values in those patients who met the criteria of MetS. Interestingly, Yegin et al. [40] reported a positive correlation between OPN concentration and leptin, total CHOL, BMI and waist circumference and suggested that OPN may be a key mediator involved in the pathogenesis of MetS.

## 5. Conclusions

In conclusion, OPN as a marker of inflammation seems to be a significant protein involved in the development of Ps and MetS. However, studies to date do not allow for a precise definition of its role in these processes.

## Figures and Tables

**Table 1:** Characteristics of the psoriatic patients and the healthy volunteers.

Characteristics	Variable	IU	Parameter	Ps Total (*N* = 107)	HVs (*N* = 38)	*p*^a^ Ps Total vs. HVs	Ps MetS (+) (*N* = 50)	Ps MetS (−) (*N* = 57)	*p*^b^ Ps MetS (+) vs. Ps MetS (−) vs. HVs	*p*^a^ Ps MetS (+) vs. Ps MetS (−)	*p*^a^ Ps MetS (+) vs. HVs	*p*^a^ Ps MetS (−) vs. HVs
Age	years		Me (IQR)	47 (37–58)	48 (45–50)	0.953	53 (43–59)	42 (35–57)	**0.007**	**0.006**	**0.044**	0.055
Obesity	BMI	kg/m^2^	Me (IQR)	27.74 (24.54–30–78)	22.95 (21.77–24.37)	**<0.001**	29.98 (26.87–32.66)	25.76 (23.14–28.07)	**<0.001**	**<0.001**	**<0.001**	**<0.001**
		normal	*n* (%)	32 (29.91)	32 (84.21)	**<0.001**	5 (10.00)	27 (47.37)	**<0.001**	**<0.001**	**<0.001**	**<0.001**
		overweight	*n* (%)	38 (35.51)	6 (15.79)		20 (40.00)	18 (31.58)				
		obese	*n* (%)	37 (34.58)	0 (0.00)		25 (50.00)	12 (21.05)				
	WHR	ratio	Me (IQR)	0.97 (0.94–1.03)	0.91 (0.86–0.93)	**<0.001**	1.00 (0.96–1.05)	0.96 (0.93–1.00)	**<0.001**	**0.001**	**<0.001**	**<0.001**
		≥1.0	*n* (%)	44 (41.12)	0 (0.00)	**<0.001**	28 (56)	16 (28.07)	**<0.001**	**0.003**	**<0.001**	**<0.001**
	Waist circumference	cm	Me (IQR)	98 (90–109)	92 (85–94)	**<0.001**	105 (98–114)	92 (87–103)	**<0.001**	**<0.001**	**<0.001**	0.095
		≥94 cm	*n* (%)	69 (64.49)	10 (26.32)	**<0.001**	43 (86.00)	26 (45.61)	**<0.001**	**<0.001**	**<0.001**	0.092
Blood pressure	Systolic	mm Hg	Me (IQR)	144 (130–152)	130 (125–130)	**<0.001**	146 (137–157)	139 (124–146)	**<0.001**	**0.004**	**<0.001**	**0.006**
		≥130	*n* (%)	82 (76.64)	23 (60.53)	0.056	43 (86.00)	39 (68.42)	**0.016**	**0.032**	**0.006**	0.567
	Diastolic	mm Hg	Me (IQR)	86 (77–92)	80 (81–83)	**0.010**	87 (78–95)	84 (77–92)	**0.020**	0.266	**0.007**	**0.048**
		≥85	*n* (%)	58 (54.21)	8 (21.05)	**<0.001**	30 (60.00)	28 (49.12)	**0.001**	0.260	**0.001**	**0.011**
Lipids	Total CHOL	mg/dL	Me (25–75%)	183 (154–208)	163 (136–185)	**0.003**	189 (164–217)	180 (151–203)	**0.005**	0.166	**0.001**	**0.044**
		>190	*n* (%)	45 (42.06)	6 (15.79)	**0.004**	24 (48.00)	21 (36.84)	**0.005**	0.243	**0.003**	**0.046**
	LDL-CHOL	mg/dL	Me (IQR)	99 (72–129)	105 (92–107)	0.668	99 (83–139)	93 (67–126)	0.403	0.159	0.963	0.441
		≥115	*n* (%)	35 (32.71)	4 (10.53)	**0.008**	18 (36.00)	17 (29.82)	**0.015**	0.497	**0.005**	**0.049**
	HDL-CHOL	mg/dL	Me (IQR)	47 (38–60)	52 (44–59)	0.291	39 (33–49)	57 (46–71)	**<0.001**	**<0.001**	**<0.001**	**0.111**
		≤40	*n* (%)	30 (28.04)	6 (15.79)	0.133	27 (54.00)	3 (5.26)	**<0.001**	**<0.001**	**0.001**	0.174
	TG	mg/dL	Me (IQR)	132 (88–182)	101 (76–138)	**0.008**	183 (156–225)	97 (76–133)	**<0.001**	**<0.001**	**<0.001**	0.799
		≥150	*n* (%)	41 (38.32)	7 (18.42)	**0.025**	38 (76.00)	3 (5.26)	**<0.001**	**<0.001**	**<0.001**	0.088
	CHOL/HDL	ratio	Me (IQR)	3.54 (2.79–4.89)	3.16 (2.50–4.08)	**0.022**	4.72 (3.52–6.03)	3.19 (2.47–3.64)	**<0.001**	**<0.001**	**<0.001**	0.603
		normal	*n* (%)	63 (58.88)	26 (68.42)	**0.004**	15 (30.00)	48 (84.21)	**<0.001**	**<0.001**	**<0.001**	0.011
		borderline	*n* (%)	20 (18.69)	12 (31.58)		14 (28.00)	6 (10.53)				
		high risk	*n* (%)	24 (22.43)	0 (0.00)		21 (42.00)	3 (5.26)				
	LDL/HDL	ratio	Me (IQR)	2.02 (1.32–2.87)	1.90 (1.66–2.36)	0.414	2.70 (1.74–3.63)	1.80 (1.12–2.15)	**<0.001**	**<0.001**	**0.001**	0.132
		normal	*n* (%)	82 (76.64)	38 (100.00)	**0.005**	31 (62.00)	51 (89.47)	**<0.001**	**0.002**	**<0.001**	**0.041**
		borderline	*n* (%)	16 (14.95)	0 (0.00)		11 (22.00)	5 (8.77)				
		high risk	*n* (%)	9 (8.41)	0 (0.00)		8 (16.00)	1 (1.75)				
	TG/HDL	ratio	Me (IQR)	2.63 (1.63–4.60)	2.02 (1.42–2.91)	**0.012**	4.70 (2.98–6.02)	1.80 (1.19–2.58)	**<0.001**	**<0.001**	**<0.001**	0.356
		above normal	*n* (%)	46 (42.99)	9 (23.68)	**0.035**	37 (74.00)	9 (15.79)	**<0.001**	**<0.001**	**<0.001**	0.487
Glucose	mg/dL		Me (IQR)	87 (82–95)	87 (83–89)	0.817	88 (82–97)	87 (81–95)	0.796	0.471	0.705	0.964
	≥100		*n* (%)	15 (14.02)	1 (2.63)	0.054	12 (24.00)	3 (5.26)	**0.002**	**0.005**	**0.013**	0.917

^a^ Mann–Whitney’s U test or χ^2^ test was used to compare continuous or categorical variables, respectively, between two groups; ^b^ Kruskal–Wallis’s H test or χ^2^ test was used to compare continuous or categorical variables, respectively, between three groups. Ps—Psoriasis; HVs—Healthy volunteers; MetS—Metabolic syndrome; BMI—Body mass index; WHR—Waist to hip ratio; total CHOL—Total cholesterol; HDL–CHOL—High–density lipoprotein cholesterol; LDL–CHOL—Low–density lipoprotein cholesterol; TG—Triglycerides; Me—Median; IQR—Interquartile range. Significant differences are in bold.

**Table 2:** Correlations between osteopontin serum concentration and characteristics of the psoriatic patients (*N* = 170).

Characteristics	Variable	IU or Category	Test ^1^	Estimate	*p*
Age		years	r	–0.158	0.056
Obesity	BMI	kg/m^2^	r	–0.010	0.916
		overweight or obese vs. normal	H	0.671	0.715
	WHR	ratio	r	–0.118	0.227
		≥1 vs. <1	Z	1.500	0.133
	Waist circumference	cm	r	–0.105	0.280
		≥94 cm vs. <94 cm	Z	1.833	0.060
Blood pressure	Systolic	mm Hg	r	–0.070	0.477
		≥130 vs. <130	Z	0.287	0.774
	Diastolic	mm Hg	r	–0.060	0.542
		≥85 vs. <85	Z	0.347	0.729
Lipids	Total CHOL	mg/dL	r	–0.274	**0.004**
		>190 vs. ≤190	Z	1.626	0.104
	LDL–CHOL	mg/dL	r	–0.114	0.244
		≥115 vs. <115	Z	1.145	0.252
	HDL–CHOL	mg/dL	r	–0.058	0.562
		≤40 vs. >40	Z	0.444	0.657
	TG	mg/dL	r	–0.250	**0.009**
		≥150 vs. <150	Z	2.554	**0.011**
	CHOL/HDL	ratio	r	–0.094	0.334
		borderline or high risk vs. normal	H	1.904	0.386
	LDL/HDL	ratio	r	–0.036	0.712
		borderline or high risk vs. normal	H	0.512	0.774
	TG/HDL	ratio	r	–0.149	0.125
		above normal vs. normal	Z	1.988	**0.047**
Glucose		mg/dL	r	–0.106	0.275
		≥100 vs. <100	Z	0.542	0.588

^1^ r—Spearman’s correlation coefficient, Z—Mann–Whitney’s test, H—Kruskal–Wallis’ test. BMI—Body mass index, WHR—Waist to hip ratio, total CHOL—Total cholesterol, HDL–CHOL—High–density lipoprotein cholesterol, LDL–CHOL—Low–density lipoprotein cholesterol, TG—Triglycerides. Significant differences are in bold.

**Table 3:** Osteopontin serum concentration vs. prevalence of metabolic syndrome criteria in the psoriatic patients.

Criterion	*n* (% of Psoriasis Total *N* = 107)	OPN Me (IQR)		*p* ^1^
		Fulfilled Criterion of Metabolic Syndrome	Not Fulfilled Criterion of Metabolic Syndrome	
Abdominal obesity	69 (64.49)	28.61 (24.16–39.58)	33.45 (27.04–46.87)	0.060
Hypertriglyceridemia (TG ≥ 150 or treatment for dyslipidemia)	45 (42.06)	27.04 (23.28–36.38)	33.07 (25.97–44.85)	**0.012**
Low HDL–CHOL (HDL–CHOL ≤ 40 or treatment for dyslipidemia)	36 (33.64)	31.54 (24.59–42.81)	31.65 (24.39–41.01)	0.690
Arterial hypertension (systolic ≥ 130 or diastolic ≥ 85 or treatment for hypertension)	90 (84.11)	31.30 (24.39–40.23)	31.69 (25.82–41.87)	0.591
Hyperglycemia (glucose ≥ 100 or treatment for type 2 diabetes)	17 (15.89)	28.61 (20.29–40.23)	31.91 (24.62 –41.30)	0.336

^1.^ Mann–Whitney’s U test was used to compare OPN serum concentration between psoriatic patients with a fulfilled and not fulfilled criterion. OPN—Osteopontin, HDL–CHOL—High–density lipoprotein cholesterol, LDL–CHOL—Low–density lipoprotein cholesterol, TG—Triglycerides, Me—Median, IQR—Interquartile range. Significant differences are in bold.

## Data Availability

The data presented in this study are available on request in the Department of Dermatology, Venereology and Pediatric Dermatology, Medical University of Lublin (Poland). The data are not publicly available due to privacy restrictions.

## Abbreviations

Ps	Psoriasis
MetS	Metabolic syndrome
OPN	Osteopontin
HVs	Healthy volunteers
CVDs	Cardiovascular diseases
TNF–α	Tumor necrosis factor α
IL	Interleukin
ELISA	Enzyme–linked immunosorbent assay
CRP	C–reactive protein
CHOL	Cholesterol
TG	Triglycerides
HDL–CHOL	High–density lipoprotein cholesterol
LDL–CHOL	Low–density lipoprotein cholesterol
VLDL	Very–low–density lipoprotein
BMI	Body mass index
WHR	Waist-to-hip ratio
